# The Role of Artificial Intelligence in the Prediction of Bariatric Surgery Complications: A Systematic Review

**DOI:** 10.7759/cureus.82461

**Published:** 2025-04-17

**Authors:** Mohamed Ahmed Hassan Mukhtar, Ahmed Umballi Babiker Ahmed, Mohammed Awad Siddig Mohammed, Nasereldeen Omer Ibrahim Omer, Dalia Saad Altom, Mohey Aldien A Elnour

**Affiliations:** 1 Emergency Medicine, Najran Armed Forces Hospital, Ministry of Defense Health Services, Najran, SAU; 2 General Surgery, Najran Armed Forces Hospital, Ministry of Defense Health Services, Najran, SAU; 3 General Surgery, Damad General Hospital, Ministry of Health, Damad, SAU; 4 General Surgery, Afif General Hospital, Afif City, SAU; 5 Family Medicine, Najran Armed Forces Hospital, Ministry of Defense Health Services, Najran, SAU; 6 General Surgery, Port Sudan Teaching Hospital, Port Sudan, SDN

**Keywords:** artificial intelligence, bariatric surgery, complications prediction, machine learning, systematic review

## Abstract

Obesity is a global health crisis, with bariatric surgery considered a highly effective intervention for sustained weight loss and resolution of associated health conditions. Despite its benefits, some patients experience postoperative complications, emphasizing the importance of accurate risk prediction. Traditional models often lack the capacity to manage complex clinical data. Artificial intelligence (AI) offers transformative potential for improving the prediction of surgical complications. This systematic review synthesizes existing research on AI's role in forecasting complications following bariatric surgery. The review followed PRISMA 2020 guidelines, with searches conducted across PubMed, Scopus, Web of Science, and IEEE Xplore for studies examining AI applications in this context. Seven retrospective cohort studies were included, and data were extracted on study design, AI algorithms, and outcomes. Risk of bias was assessed using PROBAST, and a narrative synthesis was conducted due to study heterogeneity. The included studies showed variability in AI model performance, with ensemble methods and neural networks generally performing better than traditional logistic regression. Reported area under the curve (AUC) values varied widely, with higher accuracy noted for predicting specific complications such as diabetes and leaks. Key challenges included overfitting, data imbalance, and limited generalizability, especially in deep learning models. Most studies were conducted in Sweden and the United States, utilizing large datasets that may introduce regional biases. Overall, AI shows promise in enhancing complication prediction in bariatric surgery, though methodological limitations highlight the need for prospective, multicenter validation. Future research should focus on addressing data imbalance, refining feature selection, and facilitating the clinical integration of AI through decision-support systems to improve patient care.

## Introduction and background

Obesity has emerged as a global health crisis, affecting over 650 million adults worldwide as of 2016, with prevalence rates continuing to rise alarmingly across diverse populations [1]. This chronic condition is intricately linked to life-threatening comorbidities, including type 2 diabetes, cardiovascular diseases, and certain cancers, placing immense strain on healthcare systems [2]. Bariatric surgery, encompassing procedures such as sleeve gastrectomy and Roux-en-Y gastric bypass, has proven to be the most effective intervention for sustained weight loss and comorbidity resolution. Over the past decade, the annual volume of bariatric surgeries has surged by approximately 40%, reflecting its critical role in obesity management [3].

Despite its efficacy, bariatric surgery is not without risks. Postoperative complications, ranging from anastomotic leaks, thromboembolic events, and surgical site infections to longer-term nutritional deficiencies, occur in 10-15% of patients, significantly impacting recovery, healthcare costs, and patient quality of life [4]. Accurate preoperative risk stratification and early complication prediction are paramount to mitigating adverse outcomes, optimizing patient selection, and personalizing postoperative care [5]. Traditional predictive models, reliant on logistic regression or scoring systems, often fall short due to their limited ability to process complex, high-dimensional data, such as electronic health records (EHRs), imaging, or real-time physiological metrics [6].

Artificial intelligence (AI), particularly machine learning (ML) and deep learning (DL), presents a transformative opportunity to enhance predictive accuracy [7]. By leveraging algorithms capable of identifying subtle patterns in vast datasets, AI models, including neural networks, decision trees, and ensemble methods, can integrate heterogeneous variables (e.g., preoperative biomarkers, imaging findings, and sociodemographic factors) to forecast complications with unprecedented precision. In healthcare, AI has already demonstrated success in diagnostic imaging, drug discovery, and predictive analytics, suggesting its potential to revolutionize surgical risk assessment [8].

Recent studies have explored AI’s application in predicting outcomes specific to bariatric surgery, such as 30-day readmissions, leak severity, and long-term weight regain [9-11]. However, the evidence remains fragmented, with variability in model architectures, data sources, and outcome measures. Moreover, the clinical integration of these tools requires rigorous evaluation of their validity, generalizability, and ethical implications.

This systematic review aims to synthesize existing research on AI’s role in predicting bariatric surgery complications. By analyzing studies across databases, we seek to identify gaps in current evidence, highlight best practices in model development, and provide recommendations for future research.

## Review

Methodology

Review Protocol

This systematic review was conducted in accordance with the Preferred Reporting Items for Systematic Reviews and Meta-Analyses (PRISMA) 2020 guidelines [12].

Eligibility Criteria

Studies were included if they focused on the application of AI techniques, such as machine learning, deep learning, or neural networks, for predicting postoperative complications associated with bariatric surgery. Only original research articles published in peer-reviewed journals were considered. The review included studies involving adult human subjects (aged 18 years and older) who underwent any form of bariatric surgery, including but not limited to gastric bypass, sleeve gastrectomy, or adjustable gastric banding. Studies were excluded if they did not incorporate AI models, did not focus on complication prediction, were non-English language articles, or were reviews, editorials, conference abstracts, or case reports.

Information Sources

A comprehensive literature search was conducted using electronic databases, including PubMed, Scopus, Web of Science, and IEEE Xplore. The search covered all studies published, without applying any restrictions on the start date of publication. In addition to database searches, the reference lists of relevant studies and review articles were manually screened to identify any additional eligible studies.

Search Strategy

**Figure 1:**
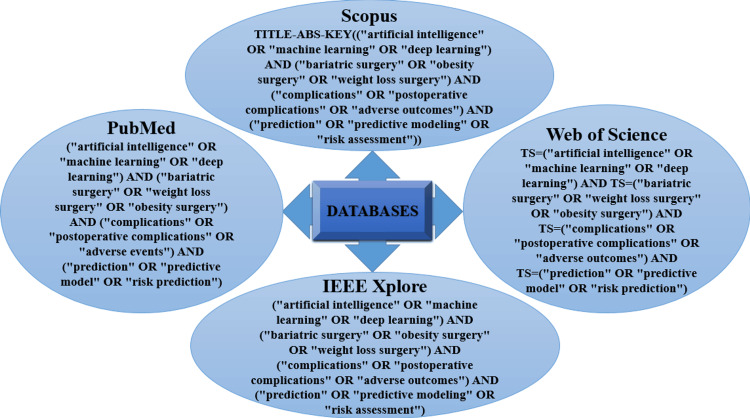
Search Strategies for Different Databases

The search strategy was designed to capture all relevant studies by combining keywords and Medical Subject Headings (MeSH) related to "artificial intelligence", "machine learning", "deep learning", "bariatric surgery", and "surgical complications". Boolean operators (AND, OR) were used to refine the search. The detailed search strings for each database are given in Figure 1.

Study Selection

All search results were imported into EndNote X9 reference management software (Clarivate, Philadelphia, Pennsylvania) for citation management, and duplicates were removed. Two independent reviewers screened the titles and abstracts of retrieved articles against the eligibility criteria. Full-text articles were then reviewed for inclusion by the same reviewers. Any disagreements were resolved through discussion or consultation with a third reviewer to ensure consensus.

Data Extraction

Data from the included studies were extracted using a standardized Microsoft Excel Sheet (Microsoft Corporation, Redmond, Washington). Extracted information included author names, year of publication, country, study design, sample size, AI algorithms, results, and key findings. The process was conducted independently by two reviewers, and discrepancies were resolved through consensus.

Risk of Bias Assessment

The risk of bias for each included study was assessed using the Prediction Model Risk of Bias Assessment Tool (PROBAST), which evaluates potential bias in studies developing or validating prediction models. This tool considers four key domains: participants, predictors, outcome, and analysis. Two independent reviewers assessed each study, and any disagreements were resolved through discussion or by consulting a third reviewer. Each domain was rated as "Low," "High," or "Unclear" risk of bias, and an overall risk of bias was determined according to PROBAST guidelines. A study was rated as having an overall "High" risk of bias if any single domain was judged to be high risk. A visual summary of the domain-level and overall judgments was presented in the form of a heatmap.

Data Synthesis

Due to anticipated heterogeneity in AI models, types of bariatric surgery, input features, and outcome definitions, a narrative synthesis was employed. The findings were grouped and summarized based on the AI methods used, the nature of predicted complications, and the overall performance of the prediction models.

Results

Study Selection Process

**Figure 2:**
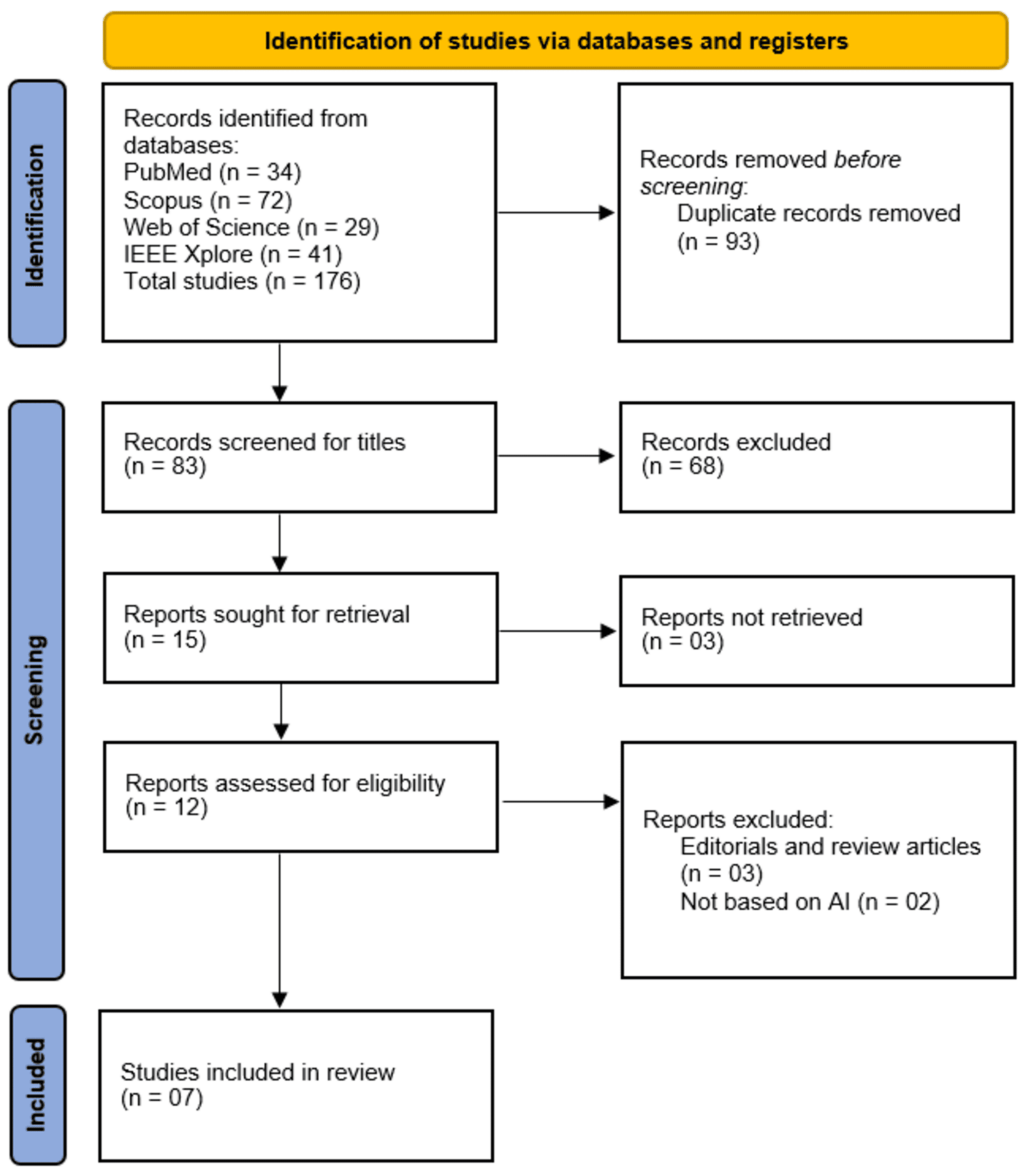
PRISMA Flow Diagram of Studies Selection PRISMA: Preferred Reporting Items for Systematic Reviews and Meta-Analyses.

The initial database search across PubMed, Scopus, Web of Science, and IEEE Xplore yielded 176 records. After removing 93 duplicate records, 83 studies underwent title and abstract screening. Of these, 68 were excluded for not meeting inclusion criteria, leaving 15 full-text articles for retrieval. Three reports could not be accessed, and 12 were assessed for eligibility. Further exclusions were made for editorials/review articles (n = 3) and studies not focused on AI (n = 2), resulting in a final inclusion of seven studies for qualitative synthesis in this systematic review (Figure 2).

Overview Included Studies

This systematic review incorporated seven retrospective cohort studies [13-19] published between 2017 and 2021, with substantial variation in sample sizes-ranging from a focused cohort of 478 patients to expansive analyses of over 4 million cases. The studies predominantly employed machine learning and deep learning approaches to predict postoperative complications following bariatric surgery, utilizing diverse algorithms including deep neural networks (DNN), support vector machines (SVM), logistic regression (LR), decision trees (DT), Bayesian networks, and ensemble methods such as random forests (RF) and AdaBoost (Table 1).

**Table 1:** Key Characteristics and Findings of Included Studies AI: artificial intelligence, ANN: artificial neural network, AUC: area under the curve, BMI: body mass index, CI: confidence interval, CNN: convolutional neural network, DBN: deep belief network, DL: deep learning, DNN: deep neural network, DT: decision tree, GERD: gastroesophageal reflux disease, HRQoL: health-related quality of life, KNN: k-nearest neighbors, LDA: linear discriminant analysis, LR: logistic regression, MLP: multi-layer perceptron, MLR: multiple logistic regression, QDA: quadratic discriminant analysis, RF: random forest, RNN: recurrent neural network, ROC: receiver operating characteristic, SMOTE: synthetic minority over-sampling technique, SVM: support vector machine, USA: United States of America, XGB: extreme gradient boosting.

Author and Publishing Year	Country	Study Design	Sample Size	AI Algorithms	Results	Key findings
Cao et al., [13] (2019)	Sweden	Retrospective study	37,811	DNN, SVM, LDA, QDA, LR, DT, KNN, MLP, and and 11 ensemble	The majority of the algorithms achieved sensitivity and accuracy of over 90%. Less than 40% sensitivity ROC is 58%.	Machine learning models showed high accuracy but low sensitivity in predicting severe bariatric surgery complications, with deep neural networks showing slight improvement.
Cao et al., [14] (2020)	Sweden	Retrospective study	44061	MLR and DBN are employed to forecast comorbidity.	AUCs for diabetes & dyslipidemia were 0.942 and 0.917, for high blood pressure and sleep apnea they were 0.891 and 0.834, and for depression they were 0.750.	Bayesian networks more accurately predicted 5-year HRQoL and comorbidities after bariatric surgery than CNN and logistic regression, showing strong performance with lower error rates.
Thomas et al., [15] (2017)	USA	Retrospective study	478	ANN	The best results for ANN-fed comorbidity-related characteristics were AUC = 0.82 & R = 0.47.	Neural networks accurately predicted long-term weight loss, with better results when short-term post-op data were included.
Cao et al., [16] (2020)	Sweden	Retrospective study	44061	CNN, MLP, and RNN	AUCs for MLP for test data were 0.84 (95% CI 0.83-0.85) & 0.54 (95% CI 0.53-0.55).	MLP and CNN showed potential for predicting serious complications after bariatric surgery, but overfitting limited their real-world performance.
Nudel et al., [17] 2020	USA	Retrospective study	436,807	XGB, ANN, and LR	For leakage prediction, ANN (AUC = 0.75), XGB, and LR performed similarly, with AUCs for venous thromboembolism of 0.65, 0.67, and 0.64.	ANN and XGB outperformed logistic regression in predicting leaks after bariatric surgery, showing promise for better risk stratification using machine learning.
Razzaghi et al., [18] (2019)	USA	Retrospective study	4000000	Bagging, RF, and AdaBoost	AdaBoost and bagging for test data had an AUC of 0.91.	Ensemble learning with feature selection and SMOTE best predicted bariatric surgery complications from imbalanced data.
Stenberg et al., [19] (2018)	Sweden	Retrospective study	44061	MLR	For Hosmer-Lemeshow, the ROC curve is 0.53, p =.056, and R2 = 0.013.	Revision surgery, older age, low BMI, large waist circumference, and dyspepsia/GERD were linked to higher risk of severe complications, but the prediction model showed low sensitivity.

The predictive performance of these models demonstrated considerable variability. While some algorithms achieved excellent discrimination, with area under the curve (AUC) values as high as 0.942 for diabetes prediction [14], others exhibited more modest performance, such as Stenberg et al. [19], which reported an AUC of 0.53 for severe complications. Notably, ensemble methods and neural networks consistently outperformed traditional statistical approaches. For instance, Razzaghi et al. [18] achieved an AUC of 0.91 using bagging and AdaBoost, while Nudel et al. [17] found that artificial neural networks (ANN) and XGBoost surpassed logistic regression in predicting anastomotic leaks (AUC 0.75 vs. 0.64).

Despite these promising results, several limitations emerged across studies. Overfitting was a recurring challenge, particularly in deep learning models, as seen in Cao et al. [16], where multilayer perceptrons (MLP) and convolutional neural networks (CNN) showed reduced generalizability despite high training accuracy. Additionally, class imbalance in outcome variables, common in surgical complication datasets, posed difficulties for model calibration, prompting some researchers to employ techniques such as SMOTE (Synthetic Minority Over-sampling Technique) to enhance predictive stability.

Geographically, the studies were concentrated in Sweden and the United States, with many leveraging large-scale national registries such as the Scandinavian Obesity Surgery Registry (SOReg) and the Metabolic and Bariatric Surgery Accreditation and Quality Improvement Program (MBSAQIP) database. This reliance on registry data, while providing robust sample sizes, also introduced potential biases related to retrospective design and variable data quality.

Included studies highlight the potential of AI in improving risk stratification for bariatric surgery complications, with advanced algorithms demonstrating superior predictive capabilities compared to conventional methods. However, the heterogeneity in model performance, along with methodological challenges such as overfitting and data imbalance, underscores the need for further validation in prospective multicenter settings before clinical implementation.

Risk of Bias Assessment Results

**Figure 3:**
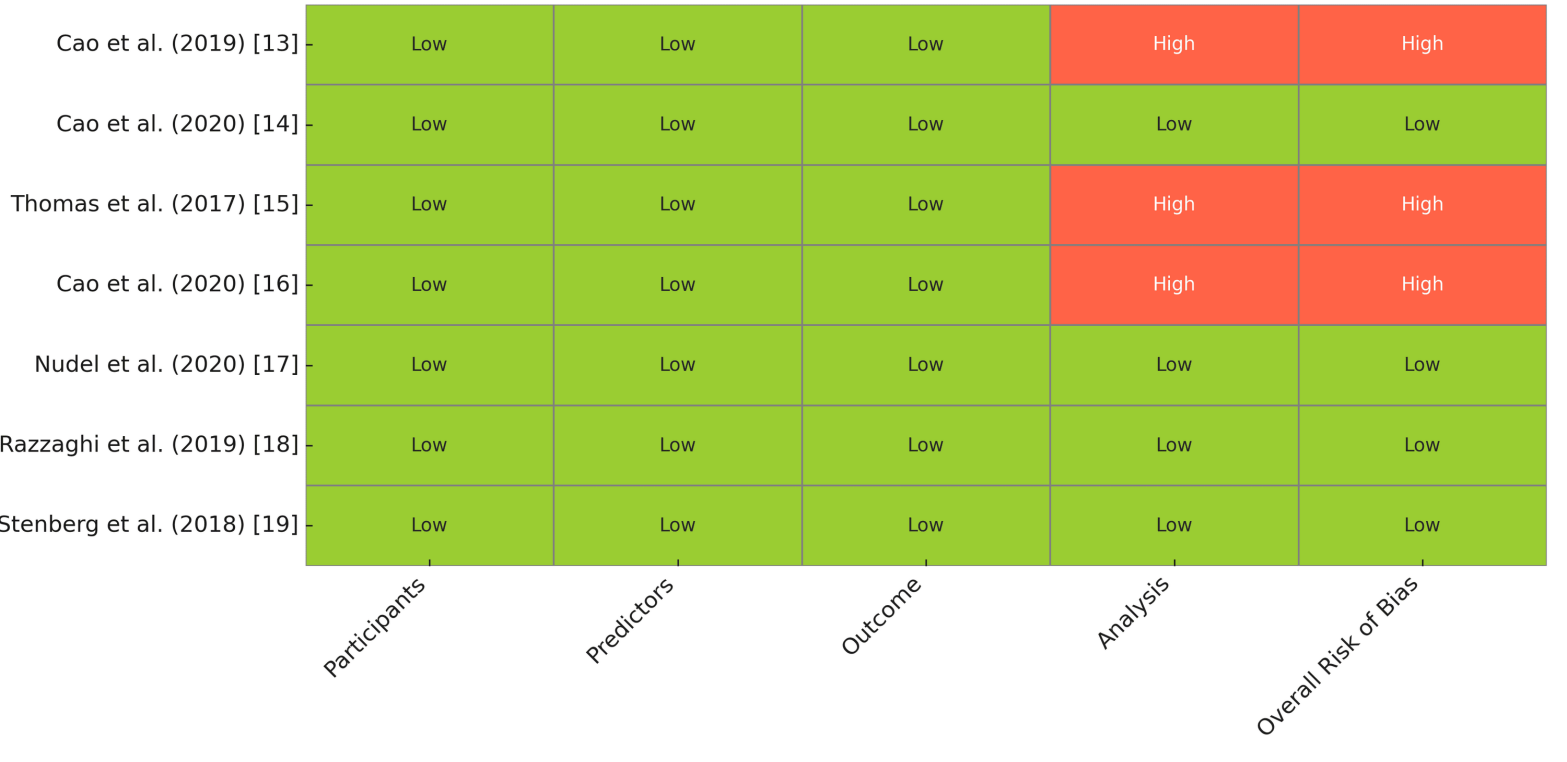
Risk of Bias Assessment for Included Studies Using PROBAST Tool

Out of the seven included studies, three were assessed as having a high overall risk of bias [13, 15, 16], while four were rated as low risk [14, 17, 18]. The domain most commonly contributing to high risk was the "Analysis" domain, primarily due to concerns related to overfitting, limited sample sizes, or lack of external validation. All studies were consistently rated as low risk in the domains of participants, predictors, and outcome, reflecting appropriate population selection, clearly defined predictors, and reliable outcome measurements. Figure 3 illustrates the domain-wise judgments across all studies, providing a comparative overview of methodological quality.

Discussion

To improve insight for prediction purposes, this study aimed to explore and describe how AI algorithms can forecast bariatric surgery issues based on the types of data collected and complications. According to the study's findings, the ANN, DBN, Ada-Boost, RF, and bagging algorithms all performed satisfactorily on this subject. For mining, RF integrates a number of DT algorithms. When used as a whole, this algorithm typically produces the best predictive results across a range of test situations. After bariatric surgery, Hsu et al. [20] found that the RF algorithm had the best predictive power for gastrointestinal bleeding. The RF outperformed other ML algorithms in Butler et al.'s [21] subsequent attempt to forecast the readmission rate following bariatric surgery, with an AUC = 0.785 (95% CI = 0.784-0.785). Weerakoon et al. [22] predicted weight reduction following bariatric surgery using several machine learning algorithms. They found that the RF model performed best for this goal, with 95% to 97% accuracy. Cao et al. [16] tried to use CNN to forecast the long-term health-related standard of life of bariatric surgery patients. After comparing this prediction accuracy with LR, they concluded that CNN gained predictive knowledge with a mean squared error that was 8% to 80% lower than LR. An ANN-related clinical decision support system (CDSS) was created by Stenberg et al. [19] to forecast the immediate side effects of gastric bypass surgery. With 98.4%, 96%, and 89.3% accuracy, respectively, the CDSS was able to forecast the difficulties that would arise after 10 days, 1 month, and 3 months. A CNN-based prediction method was employed by Cao et al. to assess type 2 diabetes recovery following bariatric surgery. With an AUC = 0.85 and 0.83, respectively, the CNN model was able to predict the pharmacological and total remissions of these patients, which was 9% to 11% better than conventional prediction methods [15].

The majority of earlier research on bariatric surgical problems was carried out in the United States and Sweden, highlighting the significance of supplemental approaches in the fight against obesity in affluent nations. Nevertheless, not much research has been done in poor nations on this subject. Recent changes in epidemiology and diet have led to an increase in the obesity problem, necessitating supportive measures like bariatric surgery. Prior research on this subject was done retrospectively. Prospective cohort investigations on various populations should be the main focus of future research to enhance data quality, including completeness, and boost mining process accuracy.

Furthermore, there has not been a thorough analysis of using machine learning techniques to forecast bariatric surgery complications. The role of AI in the early identification of potential complications or death following any gastrointestinal operation was examined by Stam et al. [23]. According to their analysis, the ML method produced a distinct performance efficiency in forecasting the complications, with an AUC that ranged from 0.50 to 0.96. Naturally, the AUC = 0.96 has significance in terms of prediction. The AI algorithms with an overall AUC of 0.84 in Henn's [24] assessment provided us with information about the good predictive performance for abdominal surgery outcomes. The AI was effective for forecasting the adverse effects of gastrointestinal surgery, according to a meta-analysis by Wang et al. [25]. The AI algorithms in the current systematic review achieved an AUC = 0.53 to 0.942, which provided us with information on performance efficiency according to the kinds of data and bariatric surgery complications. The topic makes it evident how the current study differs from previous reviews. While earlier studies have concentrated on gastrointestinal or abdominal surgery, this one particularly addresses the usefulness of machine learning in forecasting the problems of bariatric surgery. We were unable to use the meta-analysis because of the significant heterogeneity across the papers on this subject. In this condition, we narrated and examined the performance efficiency of the ML algorithms in various scenarios using the narrative synthesis of quantitative information.

According to the findings of the current review, the ensemble algorithms, which included RF, Ada-Boost, and bagging, performed better than other algorithms in terms of predicting complications, with an AUC = 0.91 on the national database. Also, in terms of generalizability and external validity, this predicted performance outperformed Cao et al.'s [13, 14, 16] research. Despite using the SMOTE technique to balance the data in some of Cao et al.'s research, we did not find significant improvements in the generalizability of the AI algorithms. The ineffectiveness of the SOReg database in forecasting bariatric surgery complications based on minority classes in a particular field and the negligible impact of SMOTE in resolving issues with imbalances in data in another are both highlighted in this topic. Therefore, in this case, we may be able to achieve greater predictive performance efficiency by employing undersampling strategies.

Undersampling approaches were likely regarded as a preferable method since they are less susceptible to overfitting than oversampling techniques, particularly when working with minority classes that have little data in large datasets. Oversampling might be better for small datasets, but overfitting is still a possibility [18]. As previously indicated, the capacity for generalization of algorithms in this circumstance may be impacted by the oversampling strategies, which increase the number of minority class instances using synthetic cases produced by AI algorithms like KNN. Furthermore, Nudel's [17] investigation showed that ANN, XG-Boost, and LR performed between 0.64 and 0.75, which is nearly sufficient for forecasting problems, without using oversampling or undersampling techniques. We advise using the ensemble techniques at the national register for prediction purposes in light of earlier research. It has been noted that the solutions to address the issues with data imbalances have significantly relied on the generalizability of databases and algorithms at the national level. More accuracy and generalizability could be obtained in some situations by using the oversampling technique, particularly if the minority class samples are reflective at this level. Otherwise, when dealing with a minority class that does not seem accurate, the undersampling method is a superior approach, and lowering the overall class would improve our predictive ability and generalizability. With an AUC of 0.82, the ANN demonstrated good performance in forecasting the complications of weight loss procedures in a single-center database. We recommend algorithms with simpler setups at this stage to carry out the prediction tasks more effectively.

We understood from the findings of earlier studies that the DBN performed better than other ML algorithms in Cao et al.'s [16] study to forecast diabetes, hypertension, dyslipidemia, and sleep apnea, respectively, with AUCs of 0.94, 0.917, 0.891, and 0.834. The SOReg registry provided us with effective predictive knowledge about these issues, even though the ML methods were unsuitable for further research because of the database's limited generalizability. With an AUC that ranged from 0.67 to 0.75, ANN, XG-Boost, and LR outperformed the rest in terms of predictive performance. Therefore, we can obtain good predictive accuracy for these issues from both ensemble and non-ensemble techniques.

The two studies on this subject used a univariate feature selection method, which is not a reliable one [15, 19]. Additionally, multivariable techniques like logistic regression help us better understand the key variables needed for prediction. This topic was examined in earlier research. Additionally, as Nudel's [17] study takes into account, determining the variables affecting bariatric surgery problems can be crucial to improving the practical application of research. To improve prognosis based on the outcome of interest, we propose a number of post-training feature ranking techniques. For instance, we propose using LIME, SHAP (Shapley Additive exPlanations), permutation feature importance, or the Relative Importance (RI) of the best algorithm obtained. Nevertheless, these methods were not taken into account in the other research on this subject. Future research must use them to improve the explainability of the algorithms with regard to bariatric surgery problems.

From an informatics perspective, the outcomes of the current investigation can be examined for therapeutic usefulness. In healthcare settings, we may create intelligent CDSSs to more accurately forecast bariatric surgery complications by utilizing the top-performing AI algorithm as a useful clinical knowledge base. According to earlier research, characteristics such as age, BMI, weight, hematocrit, height, albumin, first assistant training level, and ethnicity are crucial, particularly on a nationwide scale. When performing bariatric surgery, physicians can evaluate the patients' condition by building the CDSSs around these characteristics. With the help of such systems, they can assess people according to these characteristics and use the recommendations made by the system to help high-risk patients make better decisions for themselves and find clinical solutions, such as therapy, diagnostic, or preventive measures, to lessen surgical complications in medical settings.

Limitations

This review has several limitations. First, the included studies varied widely in sample size and data sources: some used national registries, while others relied on single-center databases, which introduced significant heterogeneity and prevented meta-analysis, necessitating a narrative synthesis instead. Additionally, complications were reported inconsistently across studies, either individually or in aggregate, further limiting comparability. Only seven studies met the inclusion criteria, reflecting the limited research on using machine learning to predict bariatric surgery complications. Although broader inclusion criteria could have increased the number of studies, the focus on complications aimed to highlight their prevention potential. Lastly, the lack of consistent reporting on predictors and their importance, especially in studies with low algorithm performance, limited our ability to draw meaningful conclusions about the most significant features.

## Conclusions

Based on the findings of earlier research on the subject, this review provided us with insight into the accuracy and efficiency of several machine learning algorithms to forecast bariatric surgery complications. We analyzed and contrasted the prediction performance efficiency of machine learning algorithms based on various databases and intraoperative complication categories using a narrative analysis of the quantitative data. According to the current review, ensemble methods have demonstrated satisfactory performance in large datasets, particularly the national register. When dealing with a single-center database, the ANN performed better than other techniques. When it came to forecasting problems like diabetes, hypertension, dyslipidemia, difficulty falling asleep, and depression, the DBN performed better. Additionally, the LR, ANN, and XG-Boost outperformed the others in terms of leakage and thrombosis prediction. We deduced from the study's findings that machine learning algorithms perform effectively and can be used as a prediction model to create a useful knowledge base for AI with the goal of reducing complications. This goal can be accomplished by providing physicians with more individualized, evidence-based clinical suggestions that are delivered by systems to help them make better clinical decisions in healthcare environments.
